# Author’s Response

**DOI:** 10.1308/003588414X13824511649373

**Published:** 2014-01

**Authors:** L Moyes

**Affiliations:** NHS Greater Glasgow and Clyde,UK

Thank you for your interest in our manuscript. You are quite correct; it should be ‘aerobic’ rather than ‘anaerobic’. We apologise for any confusion caused.

